# Photosynthetic responses of trees in high-elevation forests: comparing evergreen species along an elevation gradient in the Central Andes

**DOI:** 10.1093/aobpla/plv058

**Published:** 2015-05-22

**Authors:** José I. García-Plazaola, Roke Rojas, Duncan A. Christie, Rafael E. Coopman

**Affiliations:** 1 Departamento de Biología Vegetal y Ecología, Universidad del País Vasco UPV/EHU, Apdo. 644, E-48080 Bilbao, Spain; 2 Laboratorio de Ecofisiología para la Conservación de Bosques, Instituto de Conservación, Biodiversidad y Territorio, Facultad de Ciencias Forestales y Recursos Naturales, Universidad Austral de Chile, Casilla 567, Valdivia, Chile; 3 Laboratorio de Dendrocronología y Cambio Global, Instituto de Conservación, Biodiversidad y Territorio, Facultad de Ciencias Forestales y Recursos Naturales, Universidad Austral de Chile, Casilla 567, Valdivia, Chile; 4 Center for Climate and Resilience Research (CR)^2^, Chile

**Keywords:** High mountain plants, light-harvesting, neoxanthin, photosynthesis, xanthophylls, zeaxanthin

## Abstract

Plant growth at extremely high elevations is constrained by high daily thermal amplitude, strong solar radiation and water scarcity. These conditions are particularly harsh in the tropics, where the highest elevation treelines occur. In this environment, the maintenance of a positive carbon balance involves protecting the photosynthetic apparatus and taking advantage of any climatically favourable periods. To characterize photoprotective mechanisms at such high elevations, and particularly to address the question of whether these mechanisms are the same as those previously described in woody plants along extratropical treelines, we have studied photosynthetic responses in *Polylepis tarapacana* Philippi in the central Andes (18°S) along an elevational gradient from 4300 to 4900 m. For comparative purposes, this gradient has been complemented with a lower elevation site (3700 m) where another *Polylepis* species (*P. rugulosa* Bitter) occurs. During the daily cycle, two periods of photosynthetic activity were observed: one during the morning when, despite low temperatures, assimilation was high; and the second starting at noon when the stomata closed because of a rise in the vapour pressure deficit and thermal dissipation is prevalent over photosynthesis. From dawn to noon there was a decrease in the content of antenna pigments (chlorophyll *b* and neoxanthin), together with an increase in the content of xanthophyll cycle carotenoids. These results could be caused by a reduction in the antenna size along with an increase in photoprotection. Additionally, photoprotection was enhanced by a partial overnight retention of de-epoxized xanthophylls. The unique combination of all of these mechanisms made possible the efficient use of the favourable conditions during the morning while still providing enough protection for the rest of the day. This strategy differs completely from that of extratropical mountain trees, which uncouple light-harvesting and energy-use during long periods of unfavourable, winter conditions.

## Abstract (precis)

*Polylepis tarapacana* forms the world's highest forest, being able to grow up to 5,200 m a.s.l. At such elevations, low temperatures, high solar radiation and water scarcity severely restrict plant survival. Our study was focused on the photosynthetic responses of *Polylepis* species and how they are able to cope with such a challenging environment. We performed all measurements and samplings in their natural environment. This strategy allowed us to observe unexpected patterns of daily adjustments in photosynthetic pigments, which reflect major changes in the structure and organization of the photosynthetic apparatus.

## Introduction

One of the most challenging conditions that high-elevation plants experience is the combination of high irradiance and low temperatures. Since enzymatic reactions are sensitive to temperature, but light capture is not, this stress combination generates a severe imbalance between mechanisms' high energy absorption and low energy use through enzymatic carbon assimilation (Ball *et al*. 1991). This imbalance causes an over-excitation of the photosynthetic apparatus, which triggers the formation of reactive oxygen molecules. This reactive oxygen can provoke a large amount of oxidative damage to proteins, lipids and nucleic acids, leading to chronic photoinhibition (for a review, see Asada 2006). To counteract this effect, high mountain trees and herbs have to exacerbate the expression of protective mechanisms (Streb *et al*. 1998; Streb and Cornic 2012), thereby reaching a new equilibrium in the source/sink balance, a concept referred to as ‘photostasis’ (Öquist and Huner 2003). The photoprotective mechanisms that high mountain plants display can be classified into five major groups: (i) a decrease in light absorption by increasing reflectance through morphological/structural modifications such as trichomes or waxes (Shepherd and Griffiths 2006; Close *et al*. 2007), vertical positioning of leaves (Sanchez *et al*. 2014) or through the accumulation of anthocyanins in the upper cell layers which attenuate light reaching mesophyll cells (Williams *et al*. 2003); (ii) energy-consuming sinks' increase in metabolic activity, such as CO_2_ assimilation, plastid terminal oxidase or cyclic electron transport around photosystem I (PSI) reaction (Streb *et al*. 1998; Streb and Cornic 2012; Laureau *et al*. 2013); (iii) thermal dissipation of the excess energy absorbed by chlorophylls, a process modulated by the activity of the xanthophyll cycle that is commonly measured by the non-photochemical quenching (NPQ) of chlorophyll *a* (Chl *a*) fluorescence (for recent reviews, see Demmig-Adams *et al.* 2012; García-Plazaola *et al*. 2012); (iv) quenching through the antioxidant metabolism of reactive oxygen species generated by energy imbalance (Wildi and Lütz 1996) and (v) repair of the damage inflicted on photosystem II (PSII) reaction centres (Streb *et al*. 1997). The operation of these mechanisms has been characterized in high mountain plants in temperate regions of Europe (Ensminger *et al*. 2004; Porcar-Castell *et al*. 2012), North America (Zarter *et al*. 2006) and Australia (Ball *et al*. 1991); in these cases, a marked thermal seasonality defines a long winter and a short growing period. In these environments, the forest that extends up to the treeline is frequently made up of evergreen trees (mostly conifers in the Northern Hemisphere), which maintain light-harvesting antennae in a state primed for energy dissipation during the cold season. This mechanism is referred to as ‘sustained energy dissipation’, due to its low rate of disengagement when conditions return to their optimum state (Verhoeven 2014).

Meanwhile, treelines in the high mountain areas of North American and European temperate regions range between 1000 and 3500 m above sea level (a.s.l.), while trees in the tropical and subtropical mountains of South America are able to develop at much higher elevations (Körner 2003), with *Polylepis tarapacana* Philippi forming the highest treeline in the world at 5200 m a.s.l. (Gosling *et al*. 2009). At such high elevations, one of the most challenging environmental factors that plants have to face is the combination of large daily thermal amplitude with strong irradiance. These conditions can easily be summarized by the aphorism ‘summer every day and winter every night’ (Lüttge 2008). In the case of the South American Altiplano in the central Andes (15°–24°S), which is a semi-arid plateau located at a mean elevation of 4000 m a.s.l., the acclimation to thermal amplitude is also superimposed to seasonality (Garreaud *et al*. 2003). In the Altiplano, in contrast to extratropical mountains, the course of the seasons is mainly defined by periods of water scarcity rather than by a very cold winter. In such environments, plant species have to deal with a harsh combination of unfavourable factors including a long period of aridity, which leads to extremely low air humidity, a high proportion of short-wave radiation, low atmospheric pressure and year-round frosts (Körner 2003). Despite seasonal oscillations, the climate of the Altiplano is predominantly cold and dry. It is characterized by a dry season which corresponds to the coolest period (winter) and a warmer and wetter season (summer) when more than 80 % of the scarce total annual precipitation falls (Garreaud *et al*. 2003). Compared with higher latitudes, seasonal differences in total solar radiation are small, just 1.7 times higher in the summer than in the winter (Aceituno 1993). Under these unique environmental conditions, only trees of the genus *Polylepis* are able to subsist (González *et al*. 2002).

Species of *Polylepis* belong to the Rosaceae family, and include 28 species of small- to medium-sized evergreen trees. They grow at very high elevations in the tropical and subtropical Andes of South America from Venezuela to northern Argentina (8°N–32°S) (Kessler and Schmidt-Lebuhn 2006). Among the *Polylepis* species, *P. tarapacana* is a unique, long-lived tree that can reach over 700 years in age (Morales *et al*. 2012), and occurs throughout the semi-arid ecosystem of the Altiplano in the central Andes from 16° to 23°S between 4000 and 5200 m a.s.l., forming the world's highest elevation forests (Braun 1997). *Polylepis tarapacana* has several strategies to cope with such an unfavourable combination of environmental factors. These strategies allow this species to maintain positive assimilation rates (*P*) in both the winter and the summer (García-Núñez *et al*. 2004): *P. tarapacana* also displays frost tolerance during the cold period in addition to a super-cooling capacity during the warm season (Rada *et al*. 2001). Some of the species’ other coping strategies include xerophytic anatomical foliar traits (Toivonen *et al*. 2014) and the elevation-dependent accumulation of ultraviolet B-absorbing compounds and carotenoids (González *et al*. 2007). However, the adaptations of *P. tarapacana*'s photoprotective mechanisms (in particular the dynamics of the xanthophyll cycle) have not yet been examined.

Given the unique environmental conditions where *P. tarapacana* forests can occur, the main goal of this study was to further examine how photostasis and photosynthetic activity is modulated by the operation of photoprotective mechanisms in the world's highest forest. To achieve this objective, we studied photosynthetic responses in *P. tarapacana* along an elevation gradient (from 4337 to 4905 m a.s.l.). The study was complemented by a lower elevation *Polylepis* species growing at 3700 m a.s.l (*P. rugulosa* Bitter). We hypothesized that the photosynthetic and photoprotective mechanisms of *Polylepis* (in particular the operation of the xanthophyll cycle) at high-elevation ranges differ from those previously described for other temperate mountain systems at lower elevations, ∼1500 m a.s.l. (Zarter *et al*. 2006).

## Methods

### Study site and elevation gradient

**Figure 1.**
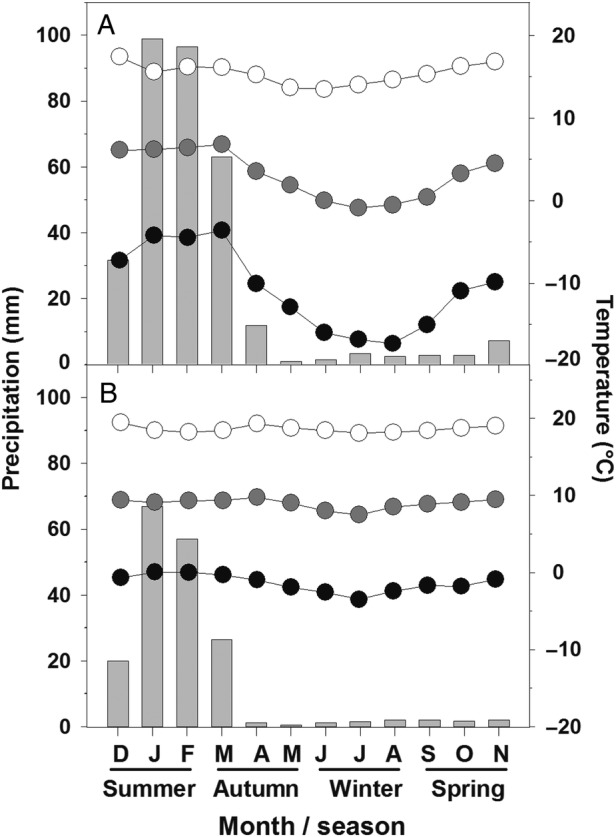
Climatic diagrams of nearby stations from the two study sites indicating monthly precipitation (grey bars), minimum (black circles), mean (grey circles) and maximum (white circles) temperatures for the period 1976–2007. (A) *Polylepis tarapacana* site and (B) *P. rugulosa* site. Total annual precipitation for each site is 322 and 182 mm, respectively.

This study was carried out in two high-elevation forest sites in the Chilean Altiplano, one of *P. rugulosa* at 3777 m a.s.l. located at its lower elevation margin (18°24′S and 69°30′W) and the other of *P. tarapacana* comprising an elevation range from 4337 to 4905 m a.s.l. (18°56′S and 69°00′W). To characterize the annual cycle of monthly precipitation, and minimum, mean and maximum temperatures for a long-term period (1976–2007), we used data from two Chilean weather service meteorological stations. These stations were located at 3545 and 4270 m a.s.l. and 25 and 16 km from the *P. rugulosa* and *P. tarapacana* sites, respectively (Fig. 1). The total annual precipitation in each forest is ∼182 and 322 mm, respectively.

During the course of measurements (10–17 h period), from 1 March through 20 March 2013, air temperature (*T*_a_, °C) and relative humidity (RH) were evaluated along the elevation gradient using dataloggers which recorded these measurements every 15 min (U23-001, Onset, MA, USA) in both the *P. rugulosa* (3777 m a.s.l.) and the *P. tarapacana* sites (4337, 4624 and 4905 m a.s.l.). Air vapour pressure deficit (VPD) was calculated, according to Murray (1967): VPD = *P*_v_ − ((RH/100) × *P*_v_), where air vapour pressure (*P*_v_) is calculated as follows: *P*_v_ = 0.611 × exp[17.27*T*_a_/(*T*_a_ + 237.3)]. Daily environmental course data from *P. rugulosa* forests were not included in the study due to technical troubles with the RH recording sensor. The diurnal course of photosynthetic photon flux density (PPFD) was characterized at both sites with microstation dataloggers recording every 10 s (H21-002 connected to two S-LIA-M003 PAR sensors, Onset, MA, USA).

### Gas exchange, Chl *a* fluorescence and optical properties

Measurements were done in March 2013, which corresponds to the beginning of the last third of the growing season for both sites. Measurements of daily course of gas exchange were performed at mid-elevation (4624 m a.s.l.) in the *P. tarapacana* site, during 3 clear days.

North-facing and sun-exposed leaves from the upper third of the canopy were measured in five different trees per day. Current season fully expanded leaves were clamped into the cuvette of an IRGA Li-6400XT with an integrated fluorescence chamber head Li-6400-40 (Li-Cor, Inc., Lincoln, NE, USA) for simultaneous measurements of gas exchange and Chl *a* fluorescence. The environmental conditions in the leaf chamber were set to be the same as the ambient conditions throughout the course of the day, and the ambient CO_2_ concentration (*C*_a_) was set at 400 µmol mol^–1^ air. Relative humidity within the leaf chamber was equilibrated with the current outside air RH every hour. The flow rate was adjusted to 300 mmol air min^–1^ to ensure that CO_2_ differentials between the reference and the sample IRGAs were >5 μmol mol^–1^ air. These deltas were achieved in all cases at 400 μmol CO_2_ mol^–1^ air. Daily course levels of PPFD were reproduced in the chamber using the ‘track ambient function’ of the Li-6400XT LED source with a 90 % red and 10 % blue light. From steady-state measurements at an ambient CO_2_ concentration (*C*_a_) of 400 μmol mol^–1^ and at natural environmental conditions, the net CO_2_ assimilation rate (*A*_N_), leaf conductance to water (*g*_l_) and sub-stomatal CO_2_ concentration (*C*_i_) were recorded. When the leaf did not cover the entire leaf cuvette surface (2 cm^2^), a digital photograph of the leaf was taken immediately after its measurement using an equal-sized foam gasket located in the same measured area as previously marked; ImageJ software (Wayne Rasband/NIH, Bethesda, MD, USA) was used to estimate the actual leaf area. Gas exchange values given by Li-6400XT were corrected using the ratio cuvette area/actual leaf area as a correction factor.

Dark-adapted fluorescence signals were measured in new, fully expanded leaves from the upper third of the plant foliage. Maximal photochemical efficiency (*F*_v_/*F*_m_) was measured in all sites before 9:00 am. The efficiency * *was measured 30 min after the leaves adapted to the dark. According to the terminology of Van Kooten and Snel (1990), minimal fluorescence (*F*_o_) was determined by applying a weak modulated light (0.4 µmol m^−2^ s^−1^) and maximal fluorescence (*F*_m_) was induced by a short pulse (0.8 s) of saturating light (∼8000 µmol m^−2^ s^−1^). The light energy partitioning at PSII was determined after 5 min of actinic light to obtain fluorescence parameters under steady-state photosynthesis. Saturating pulses were applied after steady-state photosynthesis was reached in order to determine $F_{m}^{\prime }$ and *F*_s_. Finally, the actinic light was turned off and immediately a 2 s far-red (FR) pulse was applied in order to obtain $F_{o}^{\prime }$. Hence, the saturated PSII effective photochemical quantum yield [ΦPSII], the yield of energy dissipation by antenna down-regulation [Φ(NPQ)], where NPQ refers to non-photochemical quenching, and the constitutive non-photochemical energy dissipation plus fluorescence of PSII [Φ(NO)] were calculated according to the lake model shown by Kramer *et al*. (2004). Leaf absorptance to the Li-6400 LED light was measured using a spectroradiometer EPP2000-HR (StellarNet, Inc., Tampa, FL, USA). Briefly, the method consists of measuring the incident and transmitted radiation normal to the leaf surface above and immediately below the lamina, respectively, and the reflected radiation 1 cm above the leaf by placing the sensor facing the leaf at a 45° angle from the leaf surface. The results obtained with this technique were found to be in excellent agreement with those taken with an integrating sphere (Schultz 1996; Gago *et al*. 2013).

After taking the gas exchange measurements, leaves were placed in a drying oven at 60 °C until they reached a constant weight, which was taken by estimating the leaf mass area (LMA) as dry weight/area. Leaf nitrogen (N) content per dry mass (LNC, g g^−1^ DM)] was determined in the same tissue used for LMA according to the Kjeldahl procedure (AOAC 1980).

### Photosynthetic pigments

Photosynthetic pigments were quantified in five plants per elevation; where three leaf discs (3.8 mm diameter) were collected from each plant. The same leaves used for gas exchange were also used to measure photosynthetic pigments. Samples were collected at predawn and midday, immediately frozen in liquid nitrogen and stored at −80 °C. Discs (∼30 mg fresh weight) were pulverized in a cold mortar with liquid nitrogen. To avoid acid traces, a spatula tip of CaCO_3_ was added before extraction with 1 mL of 100 % high-performance liquid chromatography (HPLC) grade acetone at 4 °C under a PPFD of 10 μmol m^−2^ s^−1^. Pigments were separated and quantified by reverse-phase HPLC (García-Plazaola and Becerril 1999), equipped with a quaternary pump with an automatic degasification system and an automatic injector. Signals from a diode matrix detector were integrated and analysed with Agilent Chem Station B.04.01 software (Agilent Technologies, Waldbronn, Germany). The chromatography was performed in a reverse-phase Spherisorb ODS-1 column (5 µm particle size; 4.6 × 250 mm, Atlantil Hilit, Waters, Ireland) and a Nova-Pack C-18 guard column (4 µm; 3.9 × 20 mm) (Waters, Ireland). The mobile phase was binary: solvent A, acetonitrile : methanol : Tris buffer (0.1 M, pH 8.0) (84 : 2 : 14); solvent B, methanol : ethyl acetate (68 : 32). Pigments were eluted using a lineal gradient of 100 % A to 100 % B within the first 12 min, followed by isocratic elution with 100 % B during the next 6 min. Absorbance was monitored at 445 nm. Retention times and response factors of Chl *a*, Chl *b*, neoxanthin (Neo), lutein (Lut), β-carotene, violaxanthin (V), anteraxanthin (A) and zeaxanthin (Z) were determined by injecting pure standards (DHI, Hoershholm, Denmark). The epoxidation state was determined as (*V* + *A*)/(*V* + *A* + *Z*).

### Statistical analysis

A one-way analysis of variance (ANOVA) was employed to determine significant differences in environmental data and descriptive foliar traits (LMA, *F*_v_/*F*_m_, absorptance, reflectance and leaf nitrogen content) for *P. tarapacana* along their elevation gradient. Gas exchange and a daily course of environmental data at a mid-elevation (4624 m a.s.l.) in the *P. tarapacana* site were compared between the morning and the afternoon (10–13 vs. 14–17 h periods).

A two-way multivariate analysis of variance on all xanthophylls and carotenes was used to evaluate differences between elevation and daily changes in pigment composition (predawn/noon). Next, we used a univariate ANOVA (following the same pattern as previously described, two-way with elevation and daily changes as fixed effects). A least-square difference (LSD) test (*P*< 0.05) was used to carry out *post hoc* analyses. Normality and homogeneity of variance were evaluated using the Shapiro–Wilk (*P* < 0.05) and Levene (*P* < 0.05) tests, respectively. When appropriate, variables were transformed to follow the former assumptions (Quinn and Keough 2006). All the statistical analyses were performed with Statistica V.7 Software (Statsoft, Tulsa, OK, USA).

## Results

**Figure 2.**
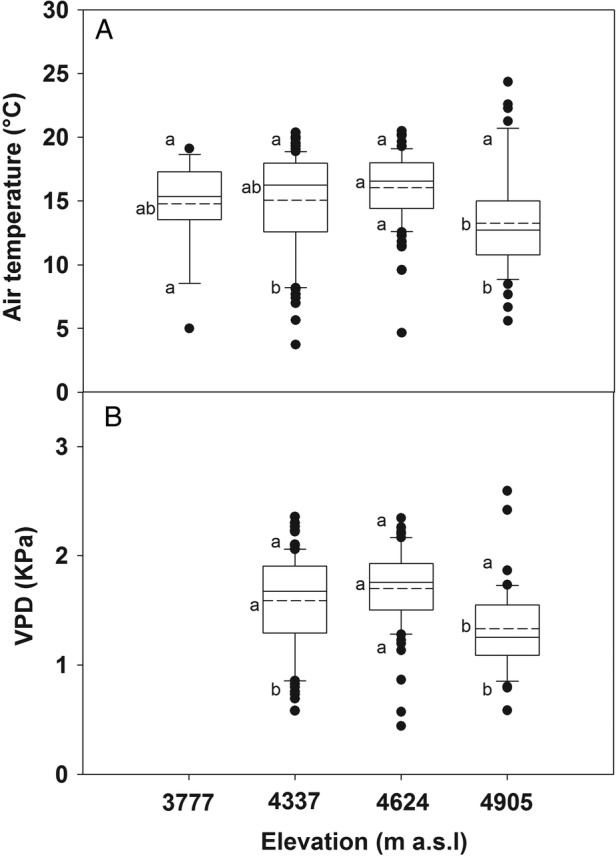
Diurnal environmental conditions during sampling and measurement days for the *P. tarapacana* and *P. rugulosa* sites. The data shown represent the climate during photosynthetic measurements (photoperiod from 10:00 to 17:00 h) from 1 March to 20 March 2013 (*n* = 19 days). Uppermost, lowest and inner continuous lines in boxplots are 25th/75th percentiles and median. Error bars are 10th/90th percentiles and black circles indicate extreme values outside these percentiles. Inner dashed lines are the mean. Different lowercase letters next to the dashed lines and error bars indicate statistically significant differences in the means and 10th/90th percentiles between elevations, as evaluated by the Fisher LSD test (*n* = 19, *P* = 0.05).

The annual variations in the climatic parameters at two nearby meteorological stations (located at 3545 and 4270 m a.s.l.) for which a long data record is available are shown in Fig. 1. Annual changes in rainfall and temperatures correspond to the typical high-elevation subtropical climate, characterized by small thermal seasonality; precipitation concentrated in the summer months and night frosts occurring year-round (Fig. 1). When comparing both stations, higher elevation in this particular tropical climate corresponds with higher precipitation, along with much lower mean temperatures. At higher elevations, thermal amplitude also increases, reaching more than 30 °C in the winter at the upper station. Meteorological data collected during the gas-exchange measurement period along the elevation gradient (Fig. 2) were consistent with these long-term measurements. Thus, the mean air temperature was 14 % lower at the higher elevation than the average of the lower elevations (*P* = 0.067). In contrast with temperature, the mean VPD decreased by 19 % at the higher elevation (*P* < 0.001). Thermal amplitude during the measurement period was remarkably 64 % higher in the *P. tarapacana* site than in the *P. rugulosa* site (*P* < 0.001). In the *P. tarapacana* site, the temperature oscillated during the study period between 18 and 9 °C. Conversely to temperature, the amplitude of VPD oscillations was 52 % higher in the lower *P. tarapacana* forest than the average of the higher elevations (*P* = 0.024).

To characterize how *P. tarapacana* leaves counteract environmental stresses associated with elevation, several descriptive foliar traits were measured in this study (LMA, *F*_v_/*F*_m_, absorptance, reflectance and leaf N content) along the elevation gradient (Table 1). However, all of them were remarkably constant in *P. tarapacana* along the elevation gradient, and no elevation-dependent trend was observed in any of the foliar traits studied (lower *P* = 0.189). Although *F*_v_/*F*_m_ did show statistical differences (*P* = 0.005), the biological meaning of this variation is negligible. Irrespective of the elevation, *P. tarapacana* leaves were thick, highly absorptive and *F*_v_/*F*_m_ was slightly lower than 0.8.

**Table 1.** Descriptive foliar traits of *P. rugulosa* and *P. tarapacana* trees grown along the elevation gradient. Leaf mass area, maximal photochemical efficiency of PSII (*F*_v_/*F*_m_), leaf absorptance and reflectance, leaves nitrogen content (LNC). Mean values ± SE are shown. Different lowercase letters indicate statistically significant differences between elevations of *P. tarapacana*, as evaluated by the Fisher LSD test (*n* = 5, *P* = 0.05).

Species	Elevation (m a.s.l.)	LMA (g m^2^)	*F*_v_/*F*_m_	Absorptance (%)	Reflectance (%)	LNC (g g^−1^)
*P. tarapacana*	4905	408 ± 6a	0.78 ± 0.01a	90 ± 0.9a	9.1 ± 1.0a	1.17 ± 0.09a
	4624	403 ± 1a	0.76 ± 0.07b	91 ± 1.9a	8.1 ± 1.7a	1.11 ± 0.04a
	4337	414 ± 11a	0.77 ± 0.05ab	87 ± 0.9a	11.0 ± 0.8a	1.05 ± 0.08a
*P. rugulosa*	3777		0.81 ± 0.03			

**Figure 3.**
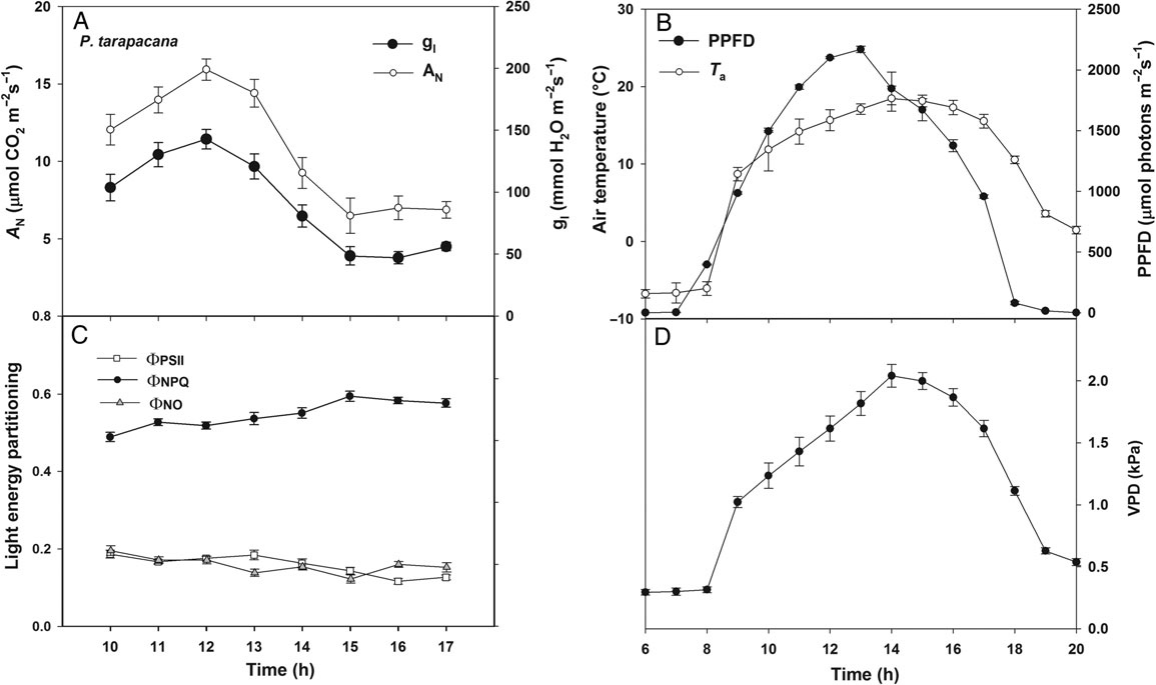
Photosynthetic daily courses of *P. tarapacana* growing at 4624 m a.s.l. (A) Net CO_2_ assimilation (*A*_N_) and leaf conductance (*g*_l_) stomatal plus cuticular conductances. (B) Photosynthetic photon flux density and air temperature (*T*_a_). (C) Light energy partitioning: PSII effective photochemical quantum yield (ΦPSII), yield of energy dissipation by antenna down-regulation [Φ(NPQ)] and constitutive non-photochemical energy dissipation plus fluorescence of PSII [Φ(NO)]. (D) Air VPD. Daily courses of gas exchange were performed during 3 clear days in five different trees per day; *n* = 15.

To gain information about the photosynthetic performance of *P. tarapacana*, daily cycles of gas exchange were studied at the mid-elevation (4624 m a.s.l.) of the *P. tarapacana* site (Fig. 3). Two periods of activity were clearly separated (10–13 vs. 14–17 h periods): morning when low VPD favoured a 99 and 83 % higher stomatal opening and carbon assimilation, respectively (Fig. 3A, higher *P* < 0.001) and afternoon, when a 24 % higher VPD (Fig. 3D; *P* < 0.001) caused stomatal closure that led to a drop in *A*_N_. This trend was confirmed by the light energy partitioning at PSII (Fig. 3D), which showed a progressive decrease in photochemistry throughout the course of the day, reaching a 19 % lower ΦPSII in the afternoon (ΦPSII; *P* < 0.001), and a parallel enhancement of 10 % in the dissipative non-photochemical heat emission (ΦNPQ; *P* < 0.001). Electron transport rates (ETRs) calculated from these data, from irradiance (Fig. 3B) and from measured leaf absorptance (Table 1), were as high as 150 µmol electrons m^−2^ s^−1^ during the highest irradiance period. According to ETR/*A*_N_ stoichiometry, ETR values are high enough to supply NADPH^+^ and ATP for the observed rate of *A*_N_ (Flexas *et al*. 2012).

**Figure 4.**
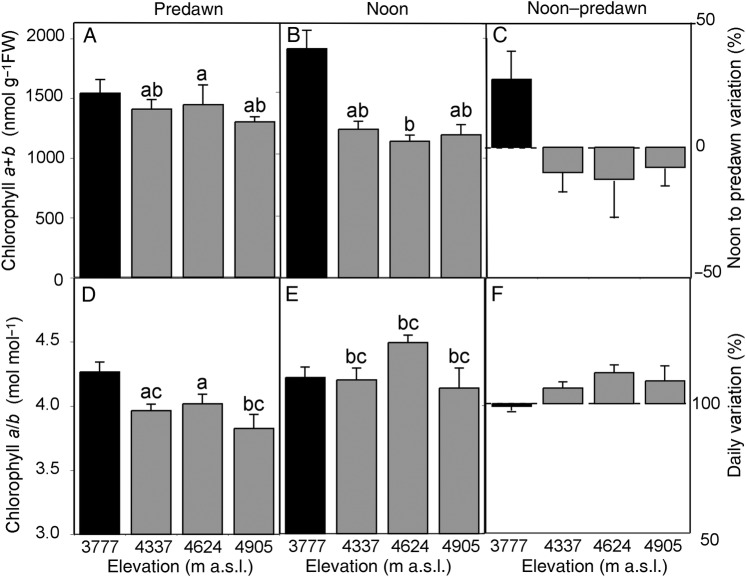
Changes in Chl *a* + *b* (A–C) and Chl *a*/*b* (D–F) in *Polylepis* species from predawn to noon along an elevation gradient. Black bars represent *P. rugulosa* and grey bars denote *P. tarapacana*. (A and D) Absolute values at predawn, (B and E) absolute values at noon and (C and F) the relative daily variation for each parameter expressed as [(noon – predawn)/noon × 100]. Details of the experimental setup are shown in Table 1. Mean values ± SE are shown. Different lowercase letters above the bars indicate statistically significant differences between elevations of *P. tarapacana* and daily variations in pigment composition, as evaluated by the Fisher LSD test (*n* = 5, *P* = 0.05).

**Figure 5.**
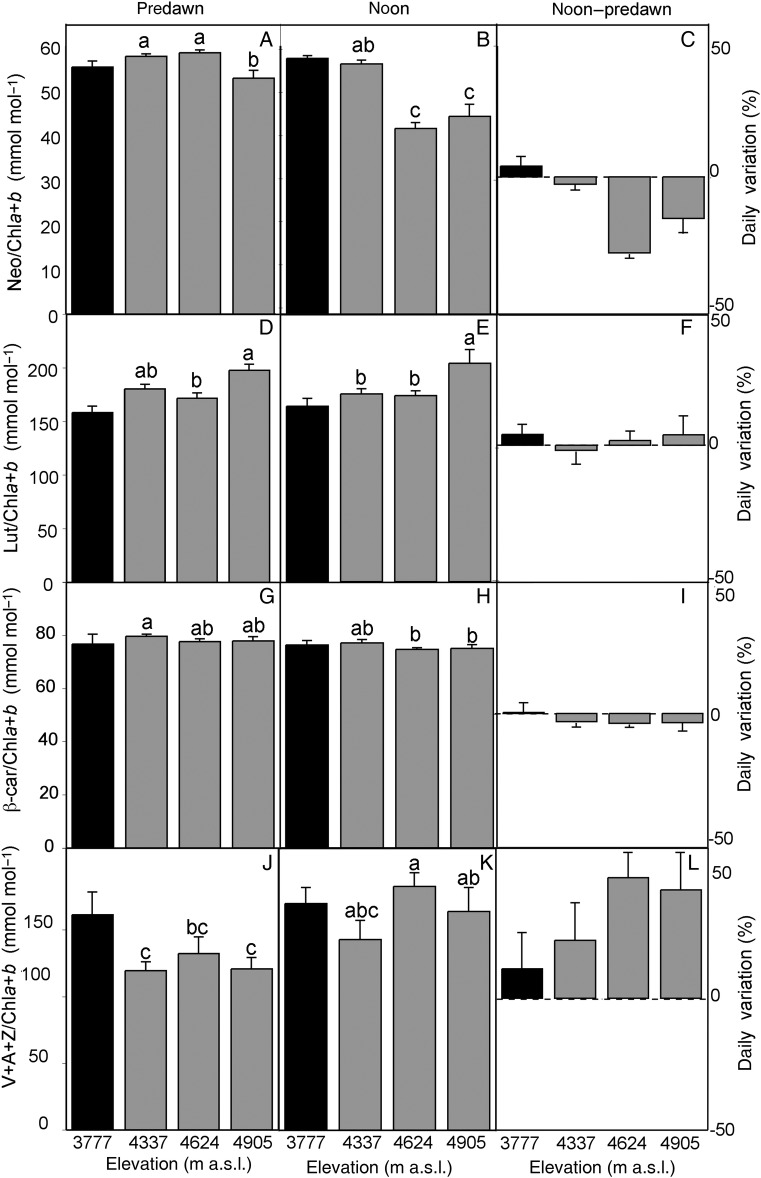
Predawn to noon changes in carotenoids to chlorophyll ratios in *Polylepis* species along an elevation gradient. Black bars represent *P. rugulosa* and grey bars denote *P. tarapacana*. (A, D, G and J) Absolute values at predawn, (B, E, H and K) absolute values at noon and (C, F, I and L) the relative daily variation for each parameter expressed as [(noon – predawn)/noon × 100]. Experimental setup details are shown in Table 1. Mean values ± SE are shown. Different lowercase letters above the bars indicate statistically significant differences between elevations of *P. tarapacana* and daily variations in pigment composition, as evaluated by the Fisher LSD test (*n* = 5, *P* = 0.05).

**Figure 6.**
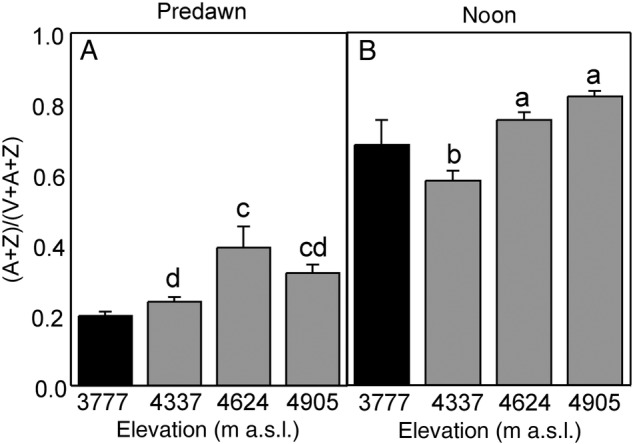
Predawn (A) and noon (B) de-epoxidation index in *Polylepis* species along an elevation gradient. Black bars represent *P. rugulosa* and grey bars indicate *P. tarapacana*. Only absolute values are shown since the relative daily variation lacks any informative sense for this parameter. Experimental setup details are shown in Table 1. Mean values ± SE are shown. Different lowercase letters above the bars indicate statistically significant differences between elevations of *P. tarapacana*, as evaluated by the Fisher LSD test (*n* = 5, *P* = 0.05).

To further understand the biochemical mechanisms responsible for the adaptation of the photosynthetic apparatus in *P. tarapacana* to the prevailing conditions at such high elevations, daily changes in pigment composition were analysed along the elevation gradient. In *P. tarapacana*, no elevation-dependent trend was observed in relation to the total chlorophyll content or the Chl *a*/*b* ratio (Fig. 4; lower *P* = 0.131), although the lower elevation species *P. rugulosa* reached a 34 % higher total chlorophyll content than that of *P. tarapaca*. However, a tendency of the net synthesis of Chl took place during the course of the first half of the day in *P. rugulosa* (24 % enhancement; *P* = 0.122), while a significant reverse trend was observed in *P. tarapacana* (16 % decrease; *P* = 0.022). As is shown by the relative changes in Chl *a*/*b*, these daily changes in the Chl content appear to be due to *de novo* synthesis of both Chl *a* and Chl *b* in *P. rugulosa* (Chl *a*/*b* did not change; *P* = 0.713), which occurred throughout the morning hours. On the other hand, the relative changes in Chl *a*/*b* in *P. tarapacana* seemed to be due to the degradation of Chl *b* (Chl *a*/*b* increased in 9 %; *P* = 0.001). When carotenoid composition was analysed in *P. tarapacana*, a different response was observed in carotenes and xanthophylls (Fig. 5). Thus, the β-carotene content did not change in response to elevation (*P* = 0.233). In contrast, the xanthophyll composition differed between elevations; for example, the highest elevation site showed the lowest Neo and highest Lut contents (higher *P* = 0.025). The Lut content was 14 % higher in *P. tarapacana* than in *P. rugulosa*, whereas *P. tarapaca* contained a 15 % smaller VAZ pool. When the carotenoid composition was compared between predawn and noon, no changes were observed in *P. rugulosa* (lower *P* = 0.282), while substantial variations were observed in *P. tarapacana*. Basically, a 16 % decrease in Neo and a 32 % enhancement in VAZ occurred (higher *P* = 0.002). These patterns were more marked in the sites at higher elevations. Apart from the higher synthesis of VAZ pigments in the two more elevated sites (4624 and 4905 m a.s.l.; higher *P* < 0.001), a concomitant 37 % higher de-epoxidation index [(*A* + *Z*)/(*V* + *A* + *Z*)] was observed at noon (Fig. 6; *P* < 0.001). Interestingly, this was accompanied by a significant overnight retention of de-epoxidized xanthophylls (*P* = 0.010), evidenced by the 52 % higher predawn values of (*A* + *Z*)/(*V* + *A* + *Z*) in these two more elevated sites.

## Discussion

Since *P. tarapacana* is an evergreen tree, its leaves have to be able to not only tolerate freezing temperatures and dry conditions throughout the year, but also must conclude the annual cycle with a positive carbon balance. In addition, during the relatively short growing season (November–April) (Solíz *et al*. 2009), photosynthesis is constrained by the high midday and afternoon VPDs in addition to the low night temperatures. All of these environmental limitations imply that *P. tarapacana*'s leaves must take advantage of this short gap of semi-favourable conditions in order to maximize carbon gain. For example, during the growing season, photosynthesis is able to proceed at temperatures close to zero (Fig. 3B) due to, among other factors, the accumulation of compatible solutes (proline and sugars) which increases its super-cooling capacity, preventing the freezing of leaves (Rada *et al*. 2001). High resistance to low-temperature photoinhibition has been described in other high-elevation plants (Germino and Smith 2000), and probably relies on a large photochemical sink (Laureau *et al*. 2013), but also on the kinetic properties of the Calvin Cycle enzymes (Streb and Cornic 2012). Therefore, despite the occurrence of extremely low night temperatures, in *P. tarapacana* the potential for carbon assimilation is maintained high, reaching maximum rates close to 15 µmol m^−2^ s^−1^ (Fig. 3A), a value which is in the upper-part of the range of photosynthetic capacity in high mountain trees and fits better with the high values reported for alpine herbs (Körner 2003) or cushion plants like the sympatric *Azorella compacta* (Kleier and Rundel 2009). This agrees with other authors (Hoch and Körner 2005; Young and León 2007) who found no evidence of carbon gain limitation along the highest treeline of *P. tarapacana*. Lower diurnal mean values of CO_2_ assimilation for the same species were found in the Sajama volcano (García-Núñez *et al*. 2004; Azócar *et al*. 2007). This site is located ∼90 km north of the *P. tarapacana* site, and has the opposite exposition; its slope faces south which makes it a shadier and colder environment. The geographical location and topographical differences between these two sites were found to be consistent with the 39 and 40 % lowest maximum PPFD and air temperature reported in both of the previous studies and this study. Further argumentation regarding differences in gas-exchange values is restricted by the lack of methodological information in García-Núñez *et al*. (2004) and Azócar *et al*. (2007).

Another remarkable foliar property observed in this study was the null phenotypic plasticity of *P. tarapacana* leaves in response to elevation, evidenced by the lack of response when several foliar attributes (LMA, foliar N, *F*_v_/*F*_m_, PPFD reflectance) were compared along the elevation gradient (Table 1). This contrasts with the general pattern described in other high mountain species in which the same parameters (LMA, leaf N, reflectance) responded differently (positively or negatively) to the elevation gradient (Meinzer *et al*. 1985; Filella and Peñuelas 1999; Taguchi and Wada 2001). The low responsiveness of *P. tarapacana*'s foliar traits to elevation gradients has been previously noted (González *et al*. 2002), although its biochemical composition has shown to be more responsive to these gradients (González *et al*. 2007). The importance of genetically determined traits in the acclimation capacity of the genus *Polylepis* has recently been addressed by Toivonen *et al*. (2014) in a common garden experiment. This study showed that, in this genus, some important leaf functional traits have been strongly selected during evolution, restricting their phenotypic plasticity.

Throughout the day, when high VPD forces stomatal closure in *P. tarapacana*, high solar radiation increases the energy excess and photochemistry is not enough to channel all of the reducing power; the re-establishment of photostasis requires the activation of alternative mechanisms able to reduce the effective absorptive cross-section of the antennae. These mechanisms are basically two: an up-regulation of NPQ and a reduction of antenna size. To evaluate the relative contribution of both mechanisms, daily changes in pigment composition were studied along the elevation gradient.

In agreement with the foliar traits shown in Table 1, the chlorophyll content was constant in *P. tarapacana*, regardless of elevation (Fig. 4A). The absence of elevation-related changes in *P. tarapacana*'s chlorophyll content was previously reported by González *et al*. (2007) who interpreted it as an adaptive mechanism that might allow this species to maintain its photosynthetic capacity along an elevation gradient. However, in the present study, the comparison between predawn and noon values, together with the more accurate analysis of photosynthetic pigments by HPLC, has allowed us to move a few steps forward in this interpretation. First, considering only the daily variations, a marked decrease in Chl *b* and Neo took place at the two higher elevations (Figs 4B and 5A). The same pattern of midday decreases in Neo has been reported in other treeline species, such as *Pinus canariensis* (Tausz *et al*. 2001). Both pigments are mostly bound to the PSI and PSII antenna proteins. Specifically, most of the Neo pool is bound in the N1 site of the lhcb1-3 proteins (Schmid 2008; Morosinotto and Bassi 2012), which constitute the outer trimeric light harvesting complexes of photosystem II (LHCII). Therefore, Neo molecules play multiple roles at structural, light-harvesting and photoprotective levels (Dall'Osto *et al*. 2007). Thus, the decrease in Neo can be plausibly explained as a result of a down-regulation of antenna size during the first part of the day. This is consistent with the fact that most of the flexibility of the light-harvesting apparatus relies on the synthesis and degradation of trimeric LHCII, while the stoichiometry of minor LHCII antenna and of LHCI to their respective reaction centres is maintained stable (Ballottari *et al*. 2007). In agreement with this hypothesis, no daily changes in Neo were observed in the lower elevation species *P. rugulosa*.

Concomitant with the decrease in the effective antenna cross section, an elevation-dependent *de novo* synthesis of the VAZ pool occurred, reaching 40 % at the site with the highest elevation (Fig. 5D). Increases throughout the course of the day in the content of VAZ pigments have been described in other high mountain plants, such as *Ranunculus glacialis* (Streb *et al*. 1997). Since there is no evidence of the additional formation of new antenna proteins or the replacement of other xanthophyll (Lut) by newly synthesized VAZ pigments, it is likely that most of these new VAZ molecules remained unbound in the thylakoid. The existence of such a pool of free xanthophylls and its antioxidant effects have been recently demonstrated (Dall'Osto *et al*. 2010; Havaux *et al*. 2007) and most likely contribute to reinforcing the antioxidant defences of *P. tarapacana* in the upper limit of its distribution. Interestingly, changes in the VAZ pool were not matched by lutein, which is also involved in photoprotection (Dall'Osto *et al*. 2006), suggesting that, under these extreme conditions, the biosynthetic β-pathway that leads to the formation of VAZ pigments prevails (Beisel *et al*. 2010). As occurred with Neo, no daily changes in VAZ pigments or Lut occurred in *P. rugulosa*, reinforcing the photoprotective interpretation of these changes in *P. tarapacana*. Nevertheless, despite all of these mechanisms, the decrease in β-carotene, independent of elevation but consistent throughout the day, may denote that these mechanisms are not enough to prevent some damage to the PSI and PSII reaction centres, as this carotenoid is basically bound to both reaction centres (Croce and van Amerongen 2011).

As another signal of stress, midday de-epoxidation of the VAZ pool, expressed as the (*A* + *Z*)/(*V* + *A* + *Z*) ratio, was also elevation dependent (Fig. 6), with the lowest value found in *P. rugulosa*. The parallel increase in energy allocated to thermal dissipation (Fig. 3C) supports the idea that the xanthophyll cycle's activity is involved in the regulation of NPQ. However, more importantly, in the most elevated sites, 30–40 % of the VAZ components were retained overnight in the de-epoxidated state (*A* and *Z*). Overnight retention of the complete VAZ pool in the de-epoxidated form is a widely described phenomenon in temperate woody plants exposed to cold winters (Demmig-Adams *et al*. 2012) and is related to the maintenance of a photoprotective, dissipative state which does not require light for its activation. This mechanism is then able to cope with the excess of energy from the earliest hours of light, when temperatures are close to the minimum value. In the case of *P. tarapacana* and *P. rugulosa*, as has been observed in other Andean plants (Bascuñán-Godoy *et al*. 2010), this retention is much lower than in the previously mentioned temperate evergreens, in which more than 80 % of the VAZ pool remained de-epoxidated overnight in the winter months (Demmig-Adams *et al*. 2012). Considering *P. tarapacana*'s high rates of photosynthesis throughout the morning, it is unlikely that this retention plays the same dissipative role that has been described in temperate evergreens (Verhoeven 2014).

## Conclusions

The only option for successful survival in such a harsh environment for *P. tarapacana* trees is to take advantage of every favourable window for carbon gain. In this sense, contrasting with other high-elevation plants, which show a more conservative strategy such as *Lobelia rhynchopetalum* (Fetene *et al*. 1997), the photosynthetic apparatus is remarkably well adapted to cope with low temperatures, and the maximum rates of carbon assimilation are remarkably high. The limiting factor is then imposed by the high VPD that occurs from noon onwards. Consequently, throughout the morning, photoprotection relies on high photochemical activity, while the activation of photoprotective mechanisms occurs in the afternoon, along with stomatal closure, and the decrease of carbon assimilation. During this period, changes in antenna pigments such as Neo and Chl *b* suggest that photostasis could be achieved by a process of antenna size readjustment, which is complemented by *de novo* synthesis of a pool of the xanthophyll cycle pigments. Some of these xanthophylls remain overnight, but their involvement in a state of sustained dissipation as occurs in temperate evergreens is unlikely considering the high rates of assimilation during the early morning. All of these mechanisms act in coordination to reduce photodamage and to allow the maintenance of a positive carbon balance in *P. tarapacana* at the world's highest treeline. This strategy contrasts with that of temperate treelines, dominated by conifers, which are characterized by the activation of a process of sustained energy dissipation during the cold season (Verhoeven 2014).

## Sources of Funding

This research was carried out with the aid of grants from the Chilean Research Council (FONDECYT 1120965 and FONDAP 15110009) awarded to D.A.C., and BFU 2010-15021 from the Spanish Ministry of Economy and Competitiveness (MINECO) and the European Regional Development Fund ERDF(FEDER) and the Basque Government (UPV/EHU-GV IT-299-07) awarded to J.I.G.-P.

## Contributions by the Authors

J.I.G.-P. was involved in research design and manuscript preparation. R.R. carried out data processing and field measurements. D.A.C. collected samples and participated in manuscript preparation. R.E.C. was involved in research design and sample collection, and participated in manuscript preparation.

## Conflict of Interest Statement

None declared.
